# Understanding how midwives employed by the National Health Service facilitate women’s alternative birthing choices: Findings from a feminist pragmatist study

**DOI:** 10.1371/journal.pone.0242508

**Published:** 2020-11-20

**Authors:** Claire Feeley, Gill Thomson, Soo Downe

**Affiliations:** 1 THRIVE Centre, ReaCH Group, University of Central Lancashire, Preston, United Kingdom; 2 MAINN Group, University of Central Lancashire, Preston, United Kingdom; Waikato Institute of Technology, NEW ZEALAND

## Abstract

UK legislation and government policy favour women’s rights to bodily autonomy and active involvement in childbirth decision-making including the right to decline recommendations of care/treatment. However, evidence suggests that both women and maternity professionals can face challenges enacting decisions outside of sociocultural norms. This study explored how NHS midwives facilitated women’s alternative physiological birthing choices–defined in this study as *‘birth choices that go outside of local/national maternity guidelines or when women decline recommended treatment of care*, *in the pursuit of a physiological birth’*. The study was underpinned by a feminist pragmatist theoretical framework and narrative methodology was used to collect professional stories of practice via self-written narratives and interviews. Through purposive and snowball sampling, a diverse sample in terms of age, years of experience, workplace settings and model of care they operated within, 45 NHS midwives from across the UK were recruited. Data were analysed using narrative thematic that generated four themes that described midwives’ processes of facilitating women’s alternative physiological births: 1. Relationship building, 2. Processes of support and facilitation, 3. Behind the scenes, 4. Birth facilitation. Collectively, the midwives were involved in a wide range of alternative birth choices across all birth settings. Fundamental to their practice was the development of mutually trusting relationships with the women which were strongly asserted a key component of safe care. The participants highlighted a wide range of personal and advanced clinical skills which was framed within an inherent desire to meet the women’s needs. Capturing what has been successfully achieved within institutionalised settings, specifically how, maternity providers may benefit from the findings of this study.

## Introduction

While international research has highlighted women want and value a normal physiological birth [1], global normal birth rates have continued to decline significantly over the past 30 years [2]. For example, the World Health Organization highlighted data from 121 countries that showed between 1990 and 2014, the global average caesarean section rate almost tripled [2]. Reversing this trend is gathering global consensus due to the associated morbidity and mortality risks to mother and baby when ‘too much medicine’ is used [2–4]. There is a concern of iatrogenic harms that could outweigh the potential benefits of life-saving interventions [2–4]. Complex sociocultural-political factors are associated with the rise in interventions, these include; technocratic [5], medicalised [6], institutionalised [7], bureaucratic [8], routine standardised care [9], risk-averse [10] and litigious cultural norms [11]. The interplay of these discourses plays out as hegemonic childbirth practices [5] that call into question the extent to which women can make their own birthing decisions, and can negatively impact on obstetricians and midwives ability to provide individualised woman-centred care [12–14]. For example, ‘guideline-centred care’ [15], describes the super-valuing of standardised clinical guidelines over women’s individual choices and professional judgement. Hyper-adherence to guidelines has been widely critiqued as contravening the basis of evidence-based medicine, where decision-making should incorporate the best available evidence *with* patients’ values/preferences *and* in the context of expert professional judgement [16–18].

The UK has strong legislation and governmental policy in support of women’s bodily autonomy, including the right to decline healthcare [19–22]. Additionally, the UK has a strong professional midwifery workforce embedded within a robust maternity care system across all birth settings (home, birth centres and hospital), with timely access to medical or paediatric care should the need arise. International evidence demonstrates that midwifery-led care [23] and midwifery-led settings [24, 25] are associated with fewer interventions, more spontaneous vaginal birth rates and positive experiences of care [23, 26]. Therefore, the UK maternity system should, in theory, generate high rates of physiologically safe normal births, and positive childbirth experiences. However, based on recent data, the UK spontaneous birth rate, without any intervention, was just 36.9% in 2016–17 [27]. Other countries, such as the Nordic countries and the Netherlands, fare far better, without compromising maternal or neonatal wellbeing [28].

Although some studies have explored women’s decision-making and experiences of alternative physiological birthing choices [29–32], few have examined the views and experiences of midwives caring for them—an important gap in the literature. To address this gap, our study explored midwives’ experiences of facilitating ‘out of guidelines care’, which we defined as ‘*women’s birth choices that go outside of local/national maternity guidelines or when women decline recommended treatment of care*, *in the pursuit of normal physiological birth’*. These choices included healthy women declining routine maternity care practices such as labour induction after 41 weeks’ gestation, or vaginal examinations to assess the progress of labour or fetal monitoring during labour. Other situations include women with medical/obstetric risk-factors who sought midwifery-led care (e.g. declining continuous electronic monitoring or induction of labour) and/or non-obstetric settings (home or birth centres).

Our recent metasynthesis [33] explored midwives’ views and experiences of caring for women making alternative birthing choices found only five studies (UK n = 3, Australia n = 1, UK, US, and New Zealand n = 1) including the views of 55 midwives. Midwives employed working within institutions (as opposed to those who were self-employed) reported a polarity of views, ranging from ‘willingly facilitative’ to ‘reluctantly accepting’ of women’s choices. Such views related to varying attitudes towards women’s autonomous decision but were also contextualised by fears and vulnerability associated with professional accountability for women’s decisions, potential workplace reprisals and/or litigation [33]. A key gap related to understanding how employed ‘willingly facilitative’ midwives managed ‘out of guideline’ requests when, at the same time, they were answerable to their employer who reinforced guideline adherence, as well as having an obligation to their professional responsibility to enable individualised woman-centred care. Therefore, this study aimed to explore the processes of facilitation of ‘out of guidelines’ care by midwives working in the National Health Service (NHS).

## Methods

### Research question

This study aimed to answer the research question: *‘How do NHS midwives self-defining as facilitative of women’s alternative birthing choices achieve their delivery of care (the what*, *how and why)*?*’*

### Study design

This study was situated within a feminist pragmatist theoretical framework that used a narrative research methodology. Pragmatism has an ontological and epistemological perspective that is rooted in human experience [34–37]; that is perceived as both an observable phenomenon and one that is constructed through sociocultural, historical, and political contexts [38, 39]. Epistemologically, pragmatists view knowledge as a tool for action [39]; a bottom-up approach in which the ontological focus is on ‘real-life problems’ with the goal of social action and change [40–42]. Feminist pragmatism is an alignment of pragmatism with broad feminist theories—both perceive that knowledge is constructed, contingent and intrinsically political [36, 43–45]. However, feminist pragmatism is situated within an ontological and epistemological philosophical movement, rather than one specific feminist sociological movement [46–48]. Seigfried [43, 47] asserts the pragmatist focus upon problematic situations that end in reconstructed (improved) situations offer feminist projects the bridge between theory and the practice of change.

Related to this study, the research question poses a feminist pragmatist inquiry—it relates to midwives’, a female-dominated profession and women’s birth choices. Women’s bodies as a site for power, control and regulation has long been discussed [49–51]; with similar issues raised for midwives as professionals [52–55]. Our earlier introduction situated the problematization of women and midwives’ autonomy, to which this study sought to generate solutions by capturing the experiences of midwives supporting women with alternative birth choices while working within an institutional context. In this study, midwives were viewed as ‘situated knowers’ [48] with the capacity to generate practice-based knowledge [56], that can be used beneficially by other maternity professionals. By using a narrative research methodology, where stories/narratives are viewed as knowledge devices [57], the midwives situated knowledge was captured and analysed via stories of professional practice.

### Reflexivity

Reflexivity is an inherent part of qualitative research where researchers articulate and consider personal biases throughout the research process [58]. By referring back to, or reflecting upon our prior positioning throughout the research process, potential blind spots or biases were challenged [58]. For transparency, we situate our prior positioning- CF and SD are midwives and GT has a background in psychology. All authors consider woman-centred care is essential to facilitate positive childbearing experiences, and that women should be the primary decision-maker. CF and SD have personal experience of organisational and personal tensions when trying to facilitate choices made by women that do not fit within standard organisational expectations. SD has also, occasionally, experienced personal and moral discomfort in the tension between always supporting women’s decision making, and professional knowledge, when the choices being requested has, in her view, a real chance of exposing the woman and/or baby to a high level of risk. GT has researched women who have experienced birth trauma and PTSD associated with poor decision-making and lack of support from healthcare professionals.

### Ethics

Ethics approval was granted by the University of Central Lancashire’s Science, Technology, Engineering, Medicine and Health ethics sub-committee on 29th November 2016 (REF 567). Participants were provided with information of the study via the participant information sheet and informed consent was obtained via signed hard copies of the consent form.

### Participants, recruitment, and setting

Inclusion criteria were: registered midwives working in the UK for the NHS who self-defined as facilitating women’s alternative birth choices as part of their regular practice. Exclusion criteria related to student midwives, independent or midwives working privately or part of a social enterprise; midwives under ‘supervised’ practice, whereby their fitness to practice had been deemed to require extra support by the UK regulatory body were also excluded.

Recruitment was carried out using purposive methods. The study was launched in January 2017 via social media, midwifery networks, professional networks and dissemination via professional bodies and organisations (e.g. Royal College of Midwives, Birthrights charity, The Practicing Midwife journal) with all participants asked to email CF direct if willing to take part. CF responded to 86 email inquiries by sending a participant information sheet to and a request to confirm their participation via email. There were two participation options; to write a self-written narrative and a follow-up interview, or an interview only. Those willing to participate were sent hard copies of the consent and demographic information form (gender, age, ethnicity, location, highest educational level attained, number of years qualified as a midwife, current employment status including department and their midwifery band) with self-addressed envelopes for return. Once the consent and demographic forms had been returned, the participant was re-contacted to make further arrangements.

Participants were recruited from across the UK and all bar one was interviewed by telephone to maximise a diverse sample. One interview was face to face as the participant lived locally to CF.

### Data collection

All data collection was carried out by CF. Where participants selected both data collection options, the self-written narratives were requested before the interview, so the contents informed the interview. Either method of data collection used a narrative approach whereby an open-ended question was posed to elicit a narrative ‘story’ response i.e. a beginning, middle and end [57]: *‘can you tell me/write about a time when you have facilitated a woman’s choices outside of the guidelines or where she declined care*?*’* Once it appeared that the participant had finished their particular story, follow-up ‘conversational’ questions [58] were asked. For example, ‘*earlier you mentioned X*, *can you tell me more about that*?*’* or *‘what did you mean by Y’*.

The self-written narrative method generated diverse accounts that ranged from ½ -7 typed pages of text (368–3411 words). All interviews were digitally recorded and took between 25–92 minutes (for those who provided a narrative account) and 23–98 minutes (those who took part in a stand-alone interview) to complete. The variation of interview length cannot be fully explained, however, CF noticed that broadly, participants with difficult or distressing accounts (mostly related to systemic issues) spoke for longer.

### Data analysis

CF transcribed all of the interviews and non-verbal utterances (i.e. sighing, giggling, laughing) were included, as were pauses and emphasised words (i.e. participants speaking loudly). Transcribing and data analysis was carried out using MAXQDA [59] a qualitative data management software tool. If respondents told more than one distinct story, the analysis focused on the first one they provided. The analysis took account of what happened, what the midwives did, how and why.

A narrative thematic approach was used based on Braun and Clarke’s [60] six-step method: familiarisation with the data, coding, searching for themes, theme review, theme redefined and named, and writing up. In keeping with narrative methods, a temporal structure was also applied [61]. This was carried out during the familiarisation stage [60] where each data was read and broad deductive categories of a chronological structure were applied i.e. situation, context, processes, experience, outcome and evaluation. Once completed, each account was read again and line-by-line inductive coding was carried out under the chronological categories [60]. Through several iterations, the coding was refined and checked against the whole dataset for consistency [60]. Searching for themes, theme reviewing, definition and naming [60] was also carried out iteratively. Working back and forth between the steps, the themes and sub-themes were merged, split, or rejected to ensure the analytical findings represented the data. During this process, the chronological deductive category labels were removed as through interpretation, new names were applied. Throughout the analytical process, ongoing discussions with GT and SD offered an intentional method to ensure consensus of theme development.

## Results

A diverse sample of n = 45 NHS midwives was recruited during January 2017-July 2019. Of these, n = 2 provided a self-written narrative only, n = 21 provided a self-written narrative and had a follow-up interview, n = 22 had an interview only (total n = 65 pieces of data).

### Demographics

Table 1 provides the demographic data for the participants. The majority were female (n = 44) and White British (n = 39) and were within the age bracket 25–34 (n = 11) and 35–44 (n = 19). The majority lived in England with distribution across all English regions. Two participants lived in Wales, and one in Northern Ireland. Just over half held degrees as the highest level of educational achievement (n = 24), with n = 17 holding additional postgraduate qualifications. The majority of participants had between 6–10 years (n = 14), or 11–20 years (n = 16) experience and two had been qualified for less than two years.

**Table 1:** Demographics of participants.

Sex	
**Female**	44
**Male**	1
	45
**Age**	
**18–24**	1
**25–34**	11
**35–44**	19
**46–54**	8
**>55**	5
	44
**Ethnicity**	
**British African-Caribbean**	1
**White British**	39
**White Welsh**	2
**White Irish**	2
**White American**	1
	45
**Region**	
**North East England**	1
**North West England**	8
**Yorkshire and Humber**	4
**East Midlands**	3
**West Midlands**	2
**Greater London**	8
**East of England**	4
**South East England**	8
**South West England**	4
**Wales**	2
**Northern Ireland**	1
**Scotland**	0
	45
**Education**	
**Diploma**	4
**Degree**	24
**Postgraduate certificate**	3
**Master's**	12
**PhD**	2
	45
**Year's qualified**	
**<2**	2
**2–5**	5
**6–10**	14
**11–20**	16
**>20**	7
**>30**	1
	45
**Employment status**	
**Full-time**	32
**Part-time**	12
**Bank**	1
	45
**Current Clinical Band**	
**Band 5**	2
**Band 6**	21
**Band 7**	14
**Band 8**	5
**Other**	3
	45
**Job role**	
**Hospital**	
**Rotational midwife**	4
**Core midwife Labour Ward**	6
**Coordinator (shift leader)**	2
**Community**	
**Community midwife**	8
**Integrated midwife (community and birth centre)**	2
**Birth centre**	5
**Homebirth Team Leader**	4
**Community Manager**	1
**Across all settings**	
**Specialist (i.e. mental health)**	5
**Supervisor**	1
**Consultant Midwife**	4
**Other**	
**Research**	1
**Education**	2
	45
**Those with additional roles**	
**Secondary**	7
**Tertiary**	3
	10
**Type of additional role**	
**Supervisor**	4
**Specialist**	1
**Research**	1
	6

In the NHS, midwives are assigned bands related to experience or job roles i.e. Band 5 is a newly qualified midwife, Band 6 are those who have completed a mentorship programme and no longer deemed ‘newly qualified (and make up the majority of the midwifery workforce). Band 7 relates to specialist roles such as mental health or team leader positions. Band 8 relates to leadership or managerial positions. In this study, all bands were represented.

### Context of care provision

Eighteen participants provided full continuity of care across the childbirth continuum and so were part of the continuum between decisions made by the woman in the antenatal period about her childbirth plans, and how these were subsequently enacted during her labour and birth. Three midwives provided continuity of antenatal care, but they were not present at the birth. Two provided some antenatal care and birth support to women they did not have a prior relationship with due to a fragmented working model of care. Twelve midwives helped women to enact their plans during the intrapartum period in a range of settings i.e. home, birth centre or hospital, but they had not been involved in the woman’s antenatal decision making about these plans. Eight midwives were in clinical roles in which they received referrals from others to provide specific care planning for women requesting out of guideline birth choices.

### Range of alternative birth choices

The participants were involved with a wide range of alternative birthing choices (see S1 Table). Some women had multiple factors that meant their choices were out of standard guideline practices. This included, for example, a woman aged 40+ years who requested a home birth after a caesarean section. These factors are not described in full for each participant, to facilitate confidentiality. Where midwives referred to providing care in a birth centre setting, it was not always clear as to whether the birth centres were free-standing (FMU) or alongside a hospital unit (AMU), therefore the generic term ‘birth centre’ is used here.

Broadly, women’s choices could be categorised in two ways. First, as healthy women declining aspects of care such as clinical care during labour, induction of labour for post-date pregnancy, prophylactic antibiotics during labour, vaginal examinations to assess the progress of labour etc. Second, women with complicated pregnancies requesting lower levels of surveillance or intervention than might be recommended in the guidelines. This included women seeking home or birth centre birth, or hospital birth without continuous electronic monitoring, and who had a previous caesarean section, twin pregnancy, a baby presenting breech, raised body mass index (BMI); or medical conditions such as diabetes, epilepsy or blood-borne viruses. A minority of midwives reported caring for women declining emergency lifesaving medical treatment.

## Findings

**Fig 1:**
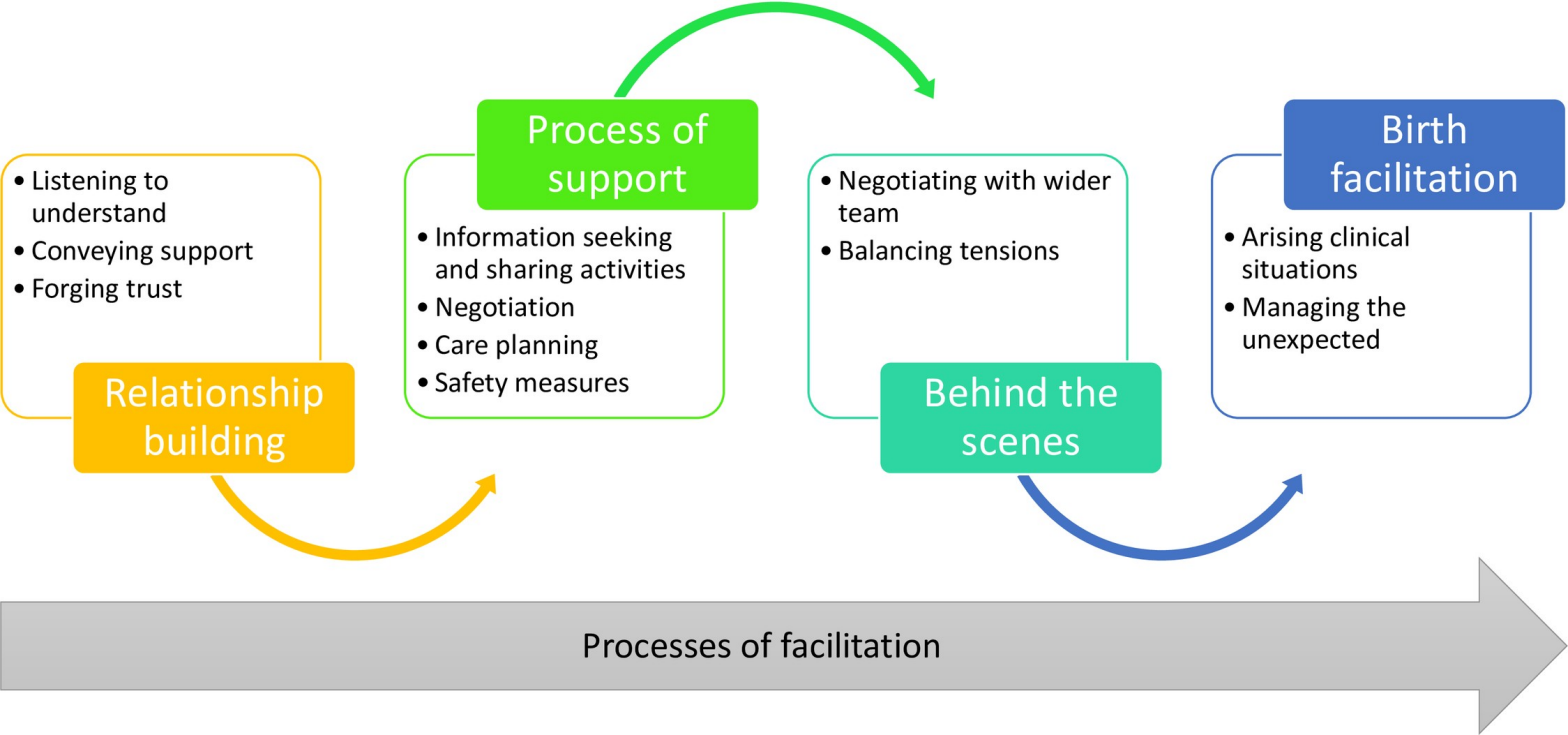
Overview of the findings.

The findings are presented within four key narrative themes and associated sub-themes; ‘*Relationship-building’*, *‘Processes’*, *‘Care planning’*, *‘Behind the scenes’*, *and ‘Birth facilitation’-* illustrated in Fig 1. Where quotes are presented, the participant pseudonym is included alongside the data source i.e. I-interview, N-self-written narrative.

### Relationship-building

A recurring issue across the participants was the prioritisation of building relationships with women; where time and effort was invested to develop and maintain positive midwife-woman relationships. Whilst this was easier for midwives working in continuity models, it was also reported by midwives working in fragmented models. For either model of practice, creating mutually trusting relationships was emphasised. Trust represented the basis of an effective working relationship and a key component of safe care. The following subthemes reflect the various components of relationship building: *‘listening to understand’*, *‘conveying attitudes of support’ and ‘forging trusting relationships*.*’*

### Listening to understand

A primary component of relationship-building was communication, specifically to understand the woman’s ‘*viewpoint*, *her history and her ethos around birth’* [Jenny]. This was referred to as *‘listening to their story and seeing how they have got to the point they are at’* [Rachel, Claire]-also illustrated by Becky:

*‘…I think is important is being able to get alongside the woman essentially and to be able to start to understand where she is coming from*. *And I think there are so many cases where women want to make choices it is really important to understand why they want to make those choices*, *and why it is they feel that is the best choice for them…’* (I)

Some of the midwives used phrases such as ‘*really listening to her’* [Rachel], depicting the importance of active and authentic listening so women felt heard, a subtle nuance articulated by Edna:

*‘…it really boils down to women feeling listened to*, *I really believe that (*.*) you know*, *listen to them*, *listen but don't just listen*, *hear what they're saying you know*?*’* (I)

Many of the midwives considered listening to women’s stories created an ‘*intimate*’ [Brigid] bond generating an empathic emotional response where they felt ‘*compelled’* [Jenny] to act. Some of the midwives attuned vividly to women’s stories of past birth trauma, demonstrating their empathic understanding. For example, Lucy used powerful language to convey the connection between the woman’s previous experience and current decision-making:

*‘She was haunted by the words spoken by the obstetric team*, *the alarm bells that echoed through the hospital corridors and couldn’t think of anywhere more frightening to birth her second child*. *Joanna told me she that was wishing to have a homebirth*, *as she felt most in control*, *comfortable and safe in her own home*, *which would therefore mean that she would have the best chance possible to labour naturally*.*’* (N)

Where women revealed stories of previous birth trauma, the midwives demonstrated understanding regarding the women’s decision-making, recognising a previous ‘*bad experience’* meant that women would *‘not want to do that again’* [Kim]. These women were often cited as having *‘a very clear idea of what they wanted’* [Jenny] in their current pregnancy, exerting agency and control in their decision-making in attempts to avoid repeated traumas.

### Conveying attitudes of support

For some midwives, conveying their support to women was an important part of building relationships. Some of the midwives expressed this as a sense of *‘responsibility’* [Alex] and *‘duty’* [Caz]:

*‘…Our job is to support her in whatever informed decisions she makes*, *as it’s her body and her birth*.*’* [Jess (N)]

Some were mindful to convey a *‘non-bullying’* [Rachel] approach, and others proactively demonstrated their support for the women’s decisions through their personal policies of *‘not saying no’* [Stella, Lauren, Anna, Kelly]. This was often carried out in the early stages of meeting the women to break down potential barriers and foster early stages of relationship building. Jenny stated:

*‘…Being told ‘you can’t’*, *‘I won’t allow’ ‘no’ can often create a communication problem that may encourage decisions based on fear of not being supported rather than a true assessment of risks and benefits*.*’* (N)

This was also expressed with an attitude that conveyed ‘*how can we help you to achieve that’* [Trish]. Communicating their support, saying yes, with a ‘how-to’ attitude appeared to be a means to disarm women who presented as defensive, ‘*prepared to fight to get the birth they want’* [Jane]. Through emphasising ‘how’, the midwives sought to convey an active demonstration of support and their investment in the woman’s experience:

*‘…I just think that makes a big difference to them (*..*) that feeling that you want them to have the experience that they want*, *and trying to see how much you can put in place to make it happen*.*’* [Trish (I)]

Some midwives recognised the potential harm of not listening to or supporting women’s decision-making. Concerns were raised of women disengaging with the services- *‘running away kicking and screaming’* [Lauren] and/or freebirthing should their needs not be met:

*‘…if we don't give these women options and don't put a support plan in place then either they won't choose to have a midwife with them or they'll just completely disengage with the care we provide*, *then it causes more problems than if we just listen to them…’* [Sam (I)]

During intrapartum situations, some midwives had less time to convey their support but managed to create time to discuss the women’s decisions whilst offering women *‘reassurance’* [Margot] that they were being listened to. It was considered *‘trickier’* [Alice] to build a relationship in fragmented care models. To convey their support, midwives reported a range of methods; verbal support, reading and respecting birth plans, and conveying agreement and understanding in a non-judgemental way.

Forging trusting relationships

A primary purpose of the midwives’ approach to listening, understanding and conveying support was to foster mutual and reciprocal trust with the women:

*‘You know*, *we wanted to be there and we wanted to support her and we wanted it to be ok but I think the real pull*, *was knowing that she trusted us*.’ *[Delilah (I)]*

Trust was perceived to reflect the two-way *‘bond’* between the mother and the midwife. In one example, Stella reported that one couple opting for a physiological breech birth explicitly demonstrated their trust in her by leaving the place of birth decision-making to her. Their explicit trust in her superseded other decisions that could have been made:

*‘We had formed quite a bond at this point*, *and Maisie and Callum [pseudonym] said openly they had trust in me*. *They were not set on a homebirth at this point*, *but said they would go wherever I was happiest–my managers had agreed I could be on call for the birth*.’ [Stella (N)]

Trust also operated both ways as midwives recognised the essential nature of women’s trust in them, which facilitated their trust in the women. For example, midwives perceived gaining a woman’s trust meant they would be more likely to accept recommendations to act, intervene and/or transfer in an emergency. Where that was established, it appeared to foster the midwives trust in the women, and greater confidence to support their decisions. For example:

*‘…I just think it's about for those women who have the more unconventional birth plans it's about making clear we're working with them*, *but that also means working with us so they do listen*, *as I say if we say actually we do need to go in*, *cos I think they're more likely to if they feel listened to and respected…*’ [Alice (N)]

In ideal situations, trust was perceived as a mutual exchange whereby the midwives’ responsibility was to support the woman’s choices and more crucially, midwives needed to demonstrate their trustworthiness, so they were ‘*judged*’ [Zoe] as trustworthy by the women. Stella reported:

*‘I think they really need to trust in the person that is there*, *that's not going to you know*, *because you and I know*, *that you could be at any birth and make something up that that*, *you can find a reason for them to be transferred’*. [Stella (I)]

As such, trust functioned as a method of safe practice, essential to the midwives’ caregiving.

## Processes of support and facilitation

Relationship building was the foundation component of caregiving. The next stage involved the midwife taking some kind of action that depended upon the complexity of the women’s decision and the midwives’ role, knowledge, or level of experience. Key processes are reported in the subthemes; *‘information seeking and sharing activities’*, *‘negotiating with women’*, *‘care planning’* and ‘*safety measures’*.

### Information seeking and sharing activities

For clinical situations that required the midwives to seek out further information, many of the midwives employed a proactive stance. This involved accessing wider information and evidence, beyond that of their local guidelines, to support women’s decision-making and to inform clinical care. This was also a method of resisting local norms where hospital policies and a standardised culture were perceived as lacking up to date evidence and limiting women’s informed choices:

*‘… I go to the RCOG*, *I go to everything like that*, *rather than just go with our hospital policy…*’ [Laura (I)].

Several methods were reported such as extensive reading [around the particular clinical situation], joining research or professional online groups to keep abreast of new research, accessing national guidelines such as NICE/RCOG, liaising with medical professionals, seeking out other hospital guidelines and accessing primary research papers. Some midwives reported that information seeking is a *‘skilled’* activity [Edna, Rachel], that requires *‘competence’* [Rachel, Laura] and was imperative to good quality care. The skills included accessing appropriate and high-quality information, the ability to understand it, and to apply it to an individual woman’s situation. The information informed discussions regarding the potential benefits and harms of particular decisions was also viewed as a professional obligation, a ‘*duty of care’* [Caz, Beatrice, Maria, Alice, Lucy] and central to providing ‘*informed choice’* [Lucy, Edna, Alice, Jenna, Jess, Catherine]. Some midwives also reported that information-seeking strategies were used to maintain clinical credibility with the women:

*‘…it is absolutely paramount (*.*) uhm (*.*) that you are up to date on current research because a lot of the women asking for out of guidelines have already done their research so they come at you often very*, *very well informed and they will eat you alive*, *you lose all clinical credibility if you (*..*)’* [Isabel (I)].

### Negotiating with women

In some situations, midwives reported negotiating care with women, usually in circumstances where women’s choices were complicated by multiple risk factors. Negotiating involved extensive discussions with women, which helped to facilitate the midwives’ deeper understanding of what women needed, or a way of determining the women’s negotiable and ‘*non-negotiable’* decisions [Trish]. For example, Trish referred to how she facilitated a woman who wanted a twin waterbirth where she had additional complications. Through determining *‘a list of her non-negotiable points*, *important points and icing on the cake wishes’* Trish identified the woman’s non-negotiable, core needs related primarily to respectful care and not the specific clinical choices:

*‘…She wanted everyone who came in her room to introduce themselves*, *no one to touch her without asking permission and all changes to the plan to be explained to her first…*’ [Trish (N)].

For some midwives, negotiations of acceptable care were to seek a compromise between institutional guidelines and the woman’s decisions. For example, Jenny supported a woman to plan for a home vaginal birth after caesarean (HVBAC), but through discussion, negotiated that transfer to hospital would be considered if active pushing in the second stage of labour took longer than one hour. In another situation, Hannah found a woman accepted a compromise of birthing on the labour ward, but with community midwives caring for her to emulate a homebirth experience. Rachel talked of the need to employ a ‘*toolbag of ideas’*. In one case, where a woman with multiple health complications had requested midwifery presence but not midwifery care, Isabel, a senior midwife, felt that she was professionally unable to be present but not to provide care. She discussed alternative services with the woman, including an independent midwife and a doula, as a method of *‘empowering the woman to find alternatives that she might not have known about before*.’

Participants negotiated within local restrictions such as institutional insurance provisions. However, such restrictions were not consistent across the participant accounts, even where the situations were similar, highlighting disparities of service provision across the NHS. For example, some midwives [Kerry, Isabel, Emily, Zoe, Stella] were enabled within their organisations to offer birth centres as alternatives for homebirths for women with risk factors. Yet, other midwives [Jenna, Beatrice, Jenny, Tracey], reported restricted access to the birth centres for such women-mandated by their organisational guidelines/insurance policies.

### Care planning

Care plans, written by midwives, but based on the woman’s decisions, were often the next activity the midwives reported in their story chronology. Care plans were a documented reflection of the discussions with women of risks, benefits, alternatives, as well as an individualised risk assessment. They were informal or formalised procedures depending on the woman’s decision, or the midwives’ workplace expectations.

Care plans were mostly viewed favourably as a tool to support women’s decision-making, a demonstration of commitment, to reduce repetitive conversations with other professionals, and a tool for advocacy in situations where women would be cared by other midwives during labour:

*‘… it's probably better to have a plan I'd say even if it's something small to ensure that what they want will be honoured*, *I think it's easier for them to go into a situation with a doctor or a midwife and all this is going on and all of a sudden they feel like they don't have a voice anymore and they can't say what they want or need (*.*)’* [Lucy (I)].

Care plans were also used as tools to support the caregiving midwives (not the participants in this study) who were reported as anxious when caring for women out of guidelines:

*‘…part of the role is about mitigating against what the women want and how the midwives will manage and cope*…’ [Hannah (I)].

Moreover, for some participants, care plans were a document that ‘*contributed to the Trust’s [institution] ability to provide vicarious liability when caring for women outside of the conventional NHS menu’* [Jenny]. As such, care plans were viewed as multi-functional, serving the needs of the Trust, health professionals and women. Overall, the care plans appeared to represent a means of legitimising the woman’s decisions, that was perceived as acceptable to other maternity professionals. Conversely, data from Katie revealed a care plan used in a coercive manner. In this situation, it was reported that management insisted the woman sign it, inferring a disclaimer.

### Safety measures

Care planning also included additional safety measures where necessary. All respondents reported that they undertook ongoing risk/benefit assessments. However, the specific activities they included were influenced by the nature of the woman’s decision, the perception of the specific risks involved, the clinical experience of the midwife and employer expectations.

Some safety measures included undertaking educational activities related to potential emergencies that may occur for the particular woman. The activities were designed to provide an opportunity for any midwife who might be on-call for the woman to practice clinical skills associated with the woman’s decision. In three situations reported, the skills training related to breech home births, and one related to a twin breech homebirth. For example:

*‘… we had several training sessions with a doll*, *pelvis and Jane Evans [independent midwife specialising in breech birth] notes*, *discussing a whole range of scenarios–what if’s*, *reasons for transfer…*’ [Stella (N)].

Some reported self-directed learning and practising a range of emergency scenarios as preparation for women’s births, in which midwives applied their existing ‘skill-set’[Amy] to new possible situations. Midwives applied their existing *‘skill-set’*[Amy] to new possible situations. This was a process that was attributed *to ‘being organised’* [Lucy] and ‘*being a forward thinker’* [Catherine]:

*‘I think I am quite an organised person and knowing that we'd been through every scenario very clearly (*.*) helped me*, *knowing we had covered all bases and planning (*.*) uhm I always set myself up for the worst-case scenario*, *which some people don't really agree with (laughs)…*’ [Lucy (I)].

In addition to individual midwives going on-call for women, some reported setting up an on-call team for the specific woman. This usually involved negotiations with fellow community midwives to ascertain who would be ‘*happy’* and ‘*comfortable*’ [Amy] and those ‘*who felt they had the skills’* [Sam] to provide intrapartum care for the woman. Whereas, midwives who felt ‘*uncomfortable*’ [Stella] or ‘*unsafe*’ [Amy] were seen as counterproductive to safe and effective care.

## Behind the scenes

So far, the majority of the findings have focused upon the midwives’ relationships with the women. However, women’s birthing decisions and the midwives’ context of practice do not exist within silos. Maternity care in the NHS includes midwifery, obstetrics, paediatrics, anaesthetists, primary care, maternity care assistants, and doulas. Some of the midwives reported negotiation strategies with the wider team. For some, this was viewed positively as a source of specialist support and help. Others midwives appeared to position themselves as a mediator between the women and medical staff. These issues are discussed in the subthemes ‘negotiating with the wider team’ and ‘balancing tensions’.

### Negotiating with the wider team

In some circumstances, respondents reported the involvement of the wider MDT, such as the obstetric, paediatric, or GP clinicians. In some situations, this was related to transferring the professional responsibility for women with moderate risk factors from consultant care back to midwifery-led care. For some, this was a straightforward process where they ‘flexibly’ worked alongside other professionals, to support the women’s decisions:

*‘…The consultants that we have working alongside us to tend to be fairly flexible as well and if a woman doesn't want something (*..*) that’s outside of the thing (*.*) they do tend to be fairly good at signing them back over to midwifery-led*. *And we'll just write you know*, *'understands that the risks are X*, *Y*, *Z and is happy to accept these risks' (*..*)’* [Claire (I)]

Other midwives perceived that ease or difficulty negotiating midwifery-led care with the MDT was dependent on the individual team member. Some obstetric doctors were viewed as more ‘supportive’ of women’s alternative decision-making than others:

*‘…but this particular Reg*[istrar] *I literally breathed a big sigh of relief*, *I never said to her [woman] that it was a game-changer…’* [Ginny (I)]

Recognising the limitations of their expertise, some of the midwives reported collaborative working to ensure the safe planning and care of women health conditions, such as epilepsy, cardiac conditions, or diabetes, or for babies anticipated to have post-birth complications. For example, Kerry reported the extensive collaboration between herself, obstetricians, neonatologists, and specialist doctors to support a woman with a blood born virus requesting a homebirth:

*‘Uhm (*..*) I think it is always the same thing*, *just the communication being really honest (*.*) and listening to them as well and making sure*, *cos (*.*) I'm not an expert in the follow-up care … (*.*) but reassuring them that I am an expert in normal birth*, *our homebirth rate was 35% so I was very confident that if things weren't going to happen we would transfer in (*..*) and definitely listening to them*, *and knowing I wasn't that expert because although we were happy to support her but there may have been specialist genuine reasons why we'd have to think of alternatives and stuff*.*’* [Kerry (I)]

### Balancing tensions

Other participants reported that negotiating with the wider MDT was problematic. Some reported that other members of staff raised concerns regarding accountability and responsibility, should an adverse outcome occur [Margot, Catherine, Susan, Ginny, Kelly]. For example, Kelly reported a negative response by a senior midwife who had been called to write a care plan for a woman having a homebirth, who had multiple obstetric complicating factors. The senior midwife was reported to be anxious, inferring she would be held responsible if anything went ‘*wrong’* during the birth. In these circumstances, some sought to provide reassurance to staff members to alleviate their concerns, and to continue facilitating women’s alternative birthing decisions. Indeed, in some cases, participants reported that dealing with concerned managers was more stressful than caring for the woman. For example, where Alice arrived at a homebirth, the woman in labour was over 42 weeks’ gestation but had not informed staff of her birth plans. Alice reported that caring for the woman was not stressful, but rather that the panic imbued by her wider team was problematic:

*‘…I didn't want her to hear everyone phoning and asking for updates*, *every half an hour 'what's going on*?*' (*..*) so that was more stressful than just looking after her*, *if I'd been left alone to look after her that would have been fine*, *it was more the stress of people going 'why isn't she in*? *why isn't she in*? *when would she have been induced*?…*’* [Alice (I)].

In some situations, the midwife participants experienced confrontations with medical colleagues who disagreed with either the woman’s decision-making or the midwife supporting her. On occasion, this was reported to lead to ‘*arguments*’ [Seana, Beatrice] or reports of comments that suggested the midwives were putting women in danger [Georgina]. As a pre-emptive measure, some midwives reported accompanying women to their consultant appointments, include for scans, or antenatal checks related to perceived problems like postmaturity. In some situations, the women had made the request, in others midwives offered it as part of routine care. In either circumstance, accompanying women appeared to serve as a method of support, advocacy and where necessary, an opportunity to directly challenge medical opinion.

## Birth facilitation

The majority of participants were involved with caring for women during the intrapartum period. The majority of women were reported to have had their birth plans fulfilled, with a small number of women reported to have changed their mind in the antenatal period. Two sub-themes describe the different experiences of midwives managing unanticipated clinical situations within planned births, or managing unexpected situations where the women’s decisions were not known to them; ‘*arising clinical situations during planned births’* and ‘*managing the unexpected’*.

### Arising clinical situations during planned births

Participants recounted how they remained aligned with women’s decision making when unexpected intrapartum events required action; the changing clinical situation required further information sharing and collaborative decision-making. In one situation, Stella reported that, despite meticulous planning for a breech birth at the local alongside birth centre, the woman concerned went into spontaneous labour and progressed rapidly at home. Stella was called to carry out a home assessment, and on arrival found the woman in advanced labour, leaving little time for transfer. A quick decision to remain at home was made in collaboration with the woman and her partner. This changing event was actioned by calling for a second midwife to assist and to carry out other necessary duties, where the outcome resulted in a safe home breech birth:

*‘… The second midwife went into another room to make calls to CDU [consultant delivery unit] and the SoM [senior midwife] on call*, *to relay plans*, *and to call for an ambulance to be on standby (part of the original agreed home plan) …’* [Stella (N)].

In another situation, Susan was supporting a woman to have a vaginal birth after caesarean (VBAC) without continuous electronic fetal monitoring (CEFM). However, during intermittent auscultation, a fetal heart abnormality was noticed and required further investigation. Susan offered to carry out CEFM for a short time of 15 minutes to ‘*ensure fetal wellbeing*’, which was accepted by the woman.:

*‘There was no resistance to this suggestion*, *the trace was reassuring and IA [intermittent auscultation] recommenced accordingly*. *The woman birthed her baby beautifully*, *without incident*, *and a physiological third stage…*.*and no VE*!! [vaginal examination*]*’ [Susan (N)].

### Managing the unexpected

In some situations, women that had not disclosed their birthing intentions, such as declining induction for postmaturity, or decline some or all intrapartum clinical observations. In these instances, some midwives perceived the lack of an antenatal care plan to be problematic, and indicative of a woman’s lack of trust that she would be supported. One example involved Zoe who was called out to a homebirth to a woman in advanced labour who did not want any midwifery care, creating tension between midwifery care and employee expectations:

*‘…uhm so I had to be very careful what I said*, *what I did and what I documented… uhm (*..*) but I did find it difficult to follow policies and protocols and be the voice for the woman as well (*…*)’* [Zoe (I)].

In contrast, Maria was in a similar situation but had a prior relationship with the woman. She reported that she *‘just sat on the sofa and watched [the woman in labour] really’*. Maria continued to demonstrate professional obligations through the writing of *‘contemporaneous notes’*, but she did not report feeling challenged by institutional policy or procedures. Other midwives also appeared to ‘go with the flow’ in unplanned situations, drawing upon their knowledge, skills and assessment of maternal and fetal wellbeing. Where observations were within normal and reassuring parameters, the midwives used this information to facilitate the woman’s decision-making. However, in a minority of situations, midwives reported women declined emergency care. While challenging for the midwives involved, Brigid reported the need to *‘meet women halfway’* and to ‘*keep your personal views out of it and be very professional’*.

## Discussion

This study describes how midwives employed by the NHS, and who self-define as facilitative of women’s out of guidelines choices, provide this care. Underpinned by a feminist pragmatist approach which sought to provide practical or practice-based evidence from the midwives working in this way, the findings have illustrated what they do, how and why. Overall, as in many other studies of maternity care, successful relationship building was central. Cultivation of mutual trust within positive relationships was significant for safe care in this situation. Safety was also reinforced by careful attention to new information and skills development that might be required to manage particular unusual situations arising out of the specific choices made, and circumstances of, some women. Respondents described how they negotiated care with women, mostly in line with a woman-centred approach. However, some negotiations related to tensions between the midwives, women’s decisions and employee expectations. Care planning and documentation was a key forward-planning process. This study also found differences in the types and quality of support from the wider multi-disciplinary team (MDT). Some were positive relationships of mutual benefit where midwives and medical professionals utilised each other’s expertise to formulate robust care plans for the women. Others reported antagonist relationships with medical staff and with other midwives in the wider teams, characterised by poor communication and a lack of trust. Finally, these findings demonstrated that most midwives were able to ‘go with the flow’ when unexpected clinical events happened, or in unexpected situations, using knowledge and skills to ensure safe woman-centred care. Overall, these findings demonstrate what has been achieved by some working within NHS maternity care settings. These successes are transferrable to other similar settings where midwifery-led care is embedded within maternity systems.

This study adds to the existing literature where quality midwife-woman relationships have been identified as key components of safe care, valued by both women [26, 62–64] and midwives. For midwives, studies have identified they value the development of positive relationships with women, and are a key source of satisfaction [65, 66], meaning in their work [67, 68], and are a source of resilience [68, 69]. The current study furthers the evidence-base by demonstrating what, how and why meaningful relationships were important for safe care in complex situations. The findings showed that through intentional ‘getting to know’ and seeking to understand the women (what), via authentic listening and a proactive approach to conveying attitudes of support (how), they fostered *mutually* trusting relationships—viewed as the foundation for safe care (why). These aspects could be argued to be the underlying mechanisms of relational-based care [23]. Others have noted that such care requires ‘emotional openness’, a willingness to be vulnerable themselves [70]. Brown’s [70] Acompañar (accompany) theory argues for mutual vulnerability, theorising that it generates a shift in power in favour of the client (woman) [70, 71]. Elements of this process were identified in the midwives’ narratives.

In the UK, maintaining up to date knowledge of evidence is a requirement for ongoing midwifery registration [72]. It is also a legal, ethical and professional requirement that midwives offer women all the information they need to make their own decision i.e. informed consent/decision-making [21, 72]. However, our findings suggest that the midwives perceived this to be unusual practice in relation to their peers which may be reflective of their ‘willingness’ to engage with women making these choices [33]. Moreover, findings from studies about women’s perspectives [32, 73, 74], and a recent legal challenge [75], suggest that not all maternity professionals are offering full information based upon women’s wants/needs.

Additionally, while complex planning in maternity care is reported across several hospital websites [76–78], and has been the subject of some discussion papers [79, 80], only one UK (unpublished) study [81] has examined the role of complex care planning concerning a range of alternative physiological birthing choices. The study used a mixed-methods design to investigate the outcomes and experiences of women seeking out of guidelines physiological birth, where care planning was carried out by a consultant midwife [81]. Outcome data of the 156 included women found that there was a lower rate of caesarean section, instrumental deliveries, and post-partum haemorrhage compared to the national averages [81]. The authors strongly advise against drawing strong conclusions due to the retrospective design and lack of matched cases [81], suggesting further research is needed. However, the qualitative data generated insights regarding the high-value women placed upon the process of shared decision-making which appeared regardless of transfer, mode, or actual place of birth [81]. This concurs with the findings in the current study, albeit from the midwives’ perspective.

This study has highlighted variations between the midwives’ experiences of working within their wider multi-disciplinary teams (MDT) and organisations. Safety in maternity care is also strongly associated with effective teamwork [82]. Even when a woman has a relationship with one midwife or obstetrician, there is always a wider maternity team behind them that requires cohesiveness for better outcomes [82]. The findings showed that midwives were often supported by the MDT to facilitate women’s decision-making, characterised by constructive relationships where expertise from either party(ies) was respected. Feasibly, positive working relationships would positively impact women’s birth experiences and the midwives’ experiences of delivering care. Conversely, a number of the midwives reported antagonist relationships with midwife peers and wider multi-disciplinary teams. Fears of accountability and responsibility, should an adverse outcome occur, echoes the wider literature related to ‘guideline-centred’ care [15], and paternalistic perspectives of women’s autonomy [83]. While supporting, facilitating and promoting physiological birth is what constitutes the role and definition of a midwife [84], studies have found that many midwives report a lack of skills and confidence working within midwifery-led, non-technical settings, with an emphasis on non-invasive care [85–87]. This could also be true for obstetrics, as interventions continue to rise, exposure to the variations of what constitutes a ‘normal’ birth is reduced [88], heightening anxiety when women opt outside of the guidelines. While midwives in this study were competent to support the women’s choices, a lack of support highlighted by conflicts and confrontations is not conducive to safe maternity care.

### Strengths and limitations

This study is the first to capture practical processes of how midwives facilitate women’s out of guideline choices, in pursuit of a physiological birth. A strength of the study is a large number of participants, and the diversity and geographical spread of the respondents, enhancing the transferability of the findings. Additionally, the two modes of data collection enhanced the study trustworthiness via triangulation. The risk of over or under interpretation of the data was minimised through explicit author positionality, reflexivity, and supervision to ensure that personal beliefs and values did not obscure important data during the analysis. There are limitations: the study only focused on midwives recruited as ‘self-defined’ facilitators of women’s alternative birthing choices and therefore does not capture the views of midwives with differing philosophies. Also, the participant claims of being facilitative of women’s choices were not verified. It is unclear whether the demographics of the included midwives (i.e. educational attainment) are representative of the wider workforce. Future research could examine this further.

## Conclusion

This qualitative study examined how NHS midwives facilitated women’s out of guideline birth choices. Based on the assumption that the midwives were ‘situated knowers’ the knowledge generated has provided practical knowledge regarding their approach, methods, and processes of supporting women in these circumstances. Due to the wide range of women’s choices this study reported, the knowledge generated has applications as heuristic knowledge which can be used by midwives more broadly within their clinical care delivery. The benefits being that the findings can be applied to most ‘out of guidelines’ clinical situations by any maternity professional. Delivering such care can be achieved by meaningful engagement with women’; through mechanisms of trust and information sharing, care plans and safety measures can be implemented to support women’s autonomous decision-making. While further research should include the views and experiences of the wider maternity team and women, these insights illuminate how full-scope midwifery care can be practised within institutional settings; with scope for transferable insights to other similar settings.

## Supporting information

S1 Table An overview of women's alternative birth choices and acronyms. (DOCX)
