# Supplementary material for: Understanding how midwives employed by the National Health Service facilitate women’s alternative birthing choices: Findings from a feminist pragmatist study
Source: PLoS One. 2020 Nov 20;15(11):e0242508. doi: 10.1371/journal.pone.0242508 (PMC7678977; doi:10.1371/journal.pone.0242508)
Supplement: S1 Table — (DOCX) [file pone.0242508.s001.docx]

# Supporting information 1: An overview of women's alternative birth choices and acronyms

| Women’s birth decisions |
| --- |
| BROAD |
| Declining vaginal examinations during labour |
| Declining postdates induction of labour (IOL) |
| Declining recommended IOL for ‘risk’ factors I.e. IVF pregnancy, >40 years old, previous caesarean |
| Declining antenatal screening/scans |
| Declining all monitoring during labour |
| Freebirth |
|  |
| HOSPITAL |
| Hospital: Declining antibiotics in labour for GBS+ or PRSOM |
| Hospital: Declining augmentation for PSROM |
| Hospital: WVBAC (with telemetry) |
| Hospital: WVBAC- declining CEFM |
| Hospital: VBAC3 |
| Hospital: declining recommended medical interventions (not emergency) |
| Hospital: declining medical interventions in emergency situations |
| Hospital: Twin waterbirth |
| Hospital: physiological third stage- PET |
| Hospital: breech births outside of guidelines |
| Hospital: waterbirth- gestational diabetes- no CEFM |
|  |
| HOMEBIRTH |
| Homebirth: >40 years old |
| Homebirth: VBAC |
| Homebirth: VBAC2 |
| Homebirth: VBAC postdates |
| Homebirth: water VBAC |
| Homebirth: grand multipara P5-P10 |
| Homebirth: PSROM>72 hours |
| Homebirth: GBS+ |
| Homebirth: diabetes (Type 1 (n=1) or GDM (n=3) |
| Homebirth: polyhydramnios |
| Homebirth: hypothyroidism |
| Homebirth: mental health needs |
| Homebirth: blood clotting disorder |
| Homebirth: epilepsy |
| Homebirth: blood-borne virus |
| Homebirth: low iron levels |
| Homebirth: raised BMI >35 |
| Homebirth: raised BMI>40 |
| Homebirth: raised BMI >50 |
| Homebirth: breech |
| Homebirth: twin breech |
| Homebirth: twin waterbirth |
| Homebirth: twins |
| Homebirth: previous history of PPH’s |
| Homebirth: previous history of shoulder dystocia |
| Homebirth: previous history of 3rd-degree tear |
| Homebirth: unusual locations |
| Homebirth: declining a recommendation of transfer for meconium liquor |
| Homebirth: declining transfer for PPH |
| Homebirth: declining transfer for stalled second stage of labour |
| Homebirth: declining transfer to hospital during prolonged third stage (>3 hours) |
|  |
| BIRTH CENTRE |
| Birth centre: outside of ‘criteria’ (unspecified) |
| Birth centre: >40 years old |
| Birth centre: blood clotting disorder |
| Birth centre: antidepressant medication |
| Birth centre: gestational diabetes |
| Birth centre: waterbirth GBS+ |
| Birth centre: raised BMI>35 |
| Birth centre: raised BMI>40 |
| Birth centre: VBAC no CEFM |
| Birth centre: breech |
|  |

### Acronyms as per the table provided

**AMU:** Alongside maternity unit (birth centre within hospital grounds)

**BMI:** Body mass index

**CEFM:** Continuous electronic fetal monitoring

**COC:** Continuity of carer

**FMU:** Free standing maternity unit (birth centre that situated away from the hospital)

**GBS/GBS+:** Group B streptococcus

**IOL:** Induction of labour

**MDT:** Multi-disciplinary team

**OU:** Obstetric unit/hospital

**P1/P2/P3** etc.: Number of births the woman has had

**PET:** Pre-eclampsia toxaemia

**PPH:** Post-partum haemorrhage

**RCM:** Royal College of Midwives

**RCOG:** Royal College of Obstetricians and Gynaecologists

**SOM:** Supervisor of Midwives

**SROM:** Spontaneous rupture of membranes

**PRSOM:** Prolonged rupture of membranes (definitions vary between 12-48 hours)

**VBAC:** Vaginal birth after caesarean section

VBAC2: Vaginal birth after two caesarean sections

VBAC3: Vaginal birth after three caesarean sections

WVBAC: Water vaginal birth after caesarean section

HVBAC: Homebirth after caesarean section

HWVBAC: Home waterbirth after caesarean section

**VE**: vaginal examination
